# Effect of Fortified *Daqu* on the Microbial Community and Flavor in Chinese Strong-Flavor Liquor Brewing Process

**DOI:** 10.3389/fmicb.2019.00056

**Published:** 2019-01-29

**Authors:** Guiqiang He, Jun Huang, Rongqing Zhou, Chongde Wu, Yao Jin

**Affiliations:** ^1^College of Light Industry, Textile and Food Engineering, Sichuan University, Chengdu, China; ^2^Key Laboratory of Leather Chemistry and Engineering, Ministry of Education, Sichuan University, Chengdu, China; ^3^National Engineering Research Center of Solid-State Manufacturing, Luzhou, China

**Keywords:** fortified *Daqu*, microbial community, fermented grain, liquor flavor, brewing process, MiSeq sequencing

## Abstract

*Daqu*, an important fermentation starter for the production of Chinese liquor, as used in the current study included traditional *Daqu* and fortified *Daqu* inoculated with *Bacillus velezensis* and *Bacillus subtilis*. To evaluate the effect of fortified *Daqu* on strong-flavor liquor production, the differences of microbial communities among three inoculation patterns of fermented grains (FG) were analyzed by the Illumina MiSeq platform. A higher relative abundance of dominant genera including *Bacillus*, *Lactococcus*, *Aspergillus*, and *Candida*, and lower relative abundance of *Lactobacillus*, were observed in FG50, in which mixed *Daqu* (traditional: fortified *Daqu* = 1: 1, w/w, 50% fortified *Daqu*) was used as the starter. Then, volatile compounds of their distillations were also examined by HS-SPME-GC-MS. The results showed that the contents of skeleton flavor components, mainly including important esters and aromatic compounds, were higher in the corresponding liquor L50, which distillated from FG50. Moreover, most esters mainly positively correlated with *Lactobacillus* and *Candida* in the bottom layer of FG fermented with 50% fortified *Daqu* (FG50-B). Aromatic compounds were strongly positively correlated with *Bacillus* and *Aspergillus* in the middle layer of FG with 50% fortified *Daqu* used (FG50-M). In particular, hexyl hexanoate showed a positive correlation with higher abundances of *Ruminococcus* in the FG with addition of 100% fortified *Daqu* (FG100). This study observed microbial compositions in the FG with fortified *Daqu* addition, and it further revealed the correlations between pivotal microbes and main flavor compounds. These results may help to develop effective strategies to regulate microbes for the brewing process and further improve the flavors of Chinese liquor.

## Introduction

Strong-flavor liquor, also called Luzhou-flavor baijiu, is the most famous Chinese liquor and has a long history (Zheng and Han, 2016; Jin et al., 2017). The annual output accounts for more than 70% of Chinese liquor as a result of its unique flavor and aroma. Strong-flavor liquor is brewed by solid-state fermentation, in which *Daqu* manufacture and grain fermentation is mainly involved (Xu et al., 2017). *Daqu*, as both an important fermenting starter and specialized raw materials, is critical for the quality of Chinese liquor (Gou et al., 2015). As is commonly known, the crude enzyme preparations and various microbes in *Daqu* are necessary for macromolecular hydrolysis and metabolism. Additionally, it contributes a large number of flavor compounds and precursors, as well as beneficial metabolites to the liquor brewing.

In general, *Daqu* was manufactured by spontaneous fermentation in an open environment. The production patterns include both simple artisanal scale and large-scale industrial processes. Until now, no matter what pattern, the production of traditional *Daqu* has mainly depended on the regulation of process parameters by skilled technicians, and the microbes mainly come from the non-sterile raw materials and the ambient environment. Therefore, the manufacture process is neither predictable nor controllable, which causes the unstable quality of the liquor.

In the last two decades, the microbial communities in different types of *Daqu*, such as Jiang-, Nong-, and Qing-xiang type, have been characterized gradually by traditional and molecular methods. For example, lactic acid bacteria were dominant in all types of *Daqu*, whereas *Bacillus* were predominant in Nong- and Jiang-xiang *Daqu* (Zhang L. et al., 2014). The fungi *Saccharomycopsis fibuligera* and *Lichtheimia ramosa* dominated in Qing- and Nong-xiang *Daqu*, while *Thermomyces lanuginosus* occurred in Jiang-xiang *Daqu* (Zheng et al., 2015). The differences of microbial communities between different types of *Daqu* are also related to the environmental conditions. For instance, moisture and acidity stresses resulted in a transition of microbial structure at thermophilic stages (Pan et al., 2016). The temperature in the *Daqu* manufacturing process is also an important factor in the composition of microbial communities (Huang et al., 2017; Xiao et al., 2017).

These results laid an important foundation for regulating the microbial community and their metabolisms in *Daqu* production. The microbial regulations based on inoculating functional strain or microflora have been applied to *Daqu* manufacturing, grain fermentation, and artificial pit mud culture (Guo et al., 2017; Sun et al., 2017; Wang P. et al., 2017). For example, *Bacillus licheniformis* and *Bacillus subtilis* have been applied to enhance the contents of organic acids in Chinese liquor (Yan et al., 2013a). A previous study showed that the microbiota’s dynamic stability was reinforced when *Bacillus*, *Pediococcus*, *Wickerhamomyces*, and *Saccharomycopsis* were inoculated to *Daqu* manufacture (Li P. et al., 2017). As for fermented grain, the diversity of bacterial community and the total ester content significantly increased by inoculating cellulase-producing bacteria (Guo et al., 2017, 2018). *Clostridium*, as one of the hexanoic acid productors, is a dominant bacteria in the aged-pit, and often inoculated into the new or young cellar to enhance the content of hexanoic acid (Zou et al., 2018).

However, it is still unclear how the microbiota of *Daqu* influences on the liquor brewing process. Thus, it is of urgent need to explore the effects of fortified *Daqu* and functional flora on the brewing process which, in turn, will help to improve the liquor quality by bioaugmentation. In this research, we attempted to provide insight into the composition of microbial communities presented in three fermented grains (FG) whereby traditional, mixed, and fortified *Daqu* were used as starter, respectively. Redundancy analysis (RDA) was applied to improve the understanding of the correlations between functional microbiota and the main flavor compounds of liquor.

## Materials and Methods

### *Daqu* Manufacture and Grain Fermentation

**FIGURE 1:**
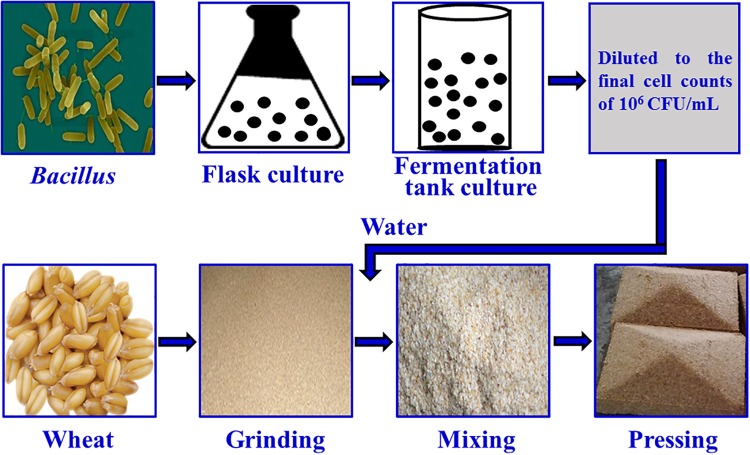
Manufacture process of fortified *Daqu*.

The fortified *Daqu* inoculated with *Bacillus velezensis* and *Bacillus subtilis* was manufactured by Xufu Brewery Co., Ltd (Yibin, China). *B*. *velezensis* and *B*. *subtilis* were isolated from *Daqu* originated from two breweries, both of which were endowed with protection owing to intangible cultural heritage. The manufacture process of *Daqu* mainly involved three procedures according to the method described previously (Zheng et al., 2012), as shown in Figure 1. First, crushed wheats were mixed with water, in which the mixture suspension with *B*. *velezensis*: *B*. *subtilis* at a ratio of 10^6^: 10^6^ cells/mL was contained, and then were pressed to shape a cuboid brick (*Daqu* bricks). Subsequently, the bricks were placed into a special room (called a Qu Fang), and cultured about 20 days according to the enterprise operation specifications. The incubation temperature of *Daqu* needs to be controlled according to the following procedure. (i) In the low-temperature incubation period, microbiota begin to grow and the temperature gradually increases, achieving 30–40°C in 2–3 days. (ii) In the high-temperature incubation period, the temperature increases by 5–8°C/day to a maximum of 55–62°C. In this phase, the relative humidity of Qu Fang is up to 90%, and doors and windows are properly opened to ventilate and lower the humidity. (iii) In the aroma-creating period, the temperature and relative humidity slowly decrease, and the aroma compounds are accumulated by the metabolic conversion. Lastly, *Daqu* were piled up to another Qu Fang, and stored more than 3 months to maturation. *Daqu* manufactured in the same batch without inoculation was used as control (traditional *Daqu*).

**FIGURE 2:**
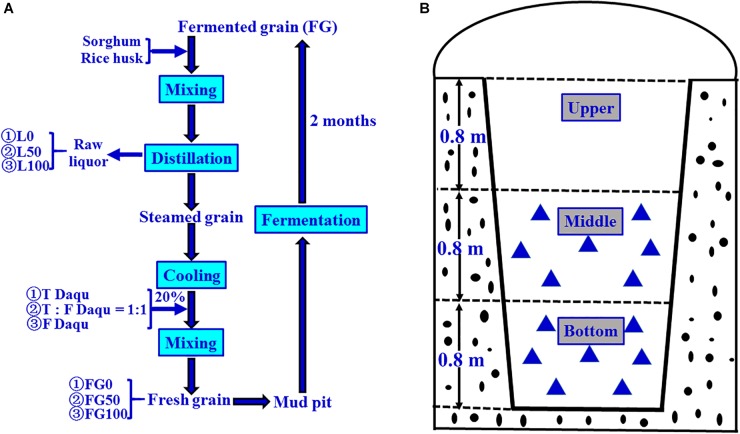
Schematic diagram of brewing process for Chinese strong-flavor liquor **(A)** and sampling method **(B)**. Different proportions (0, 50, and 100%) of fortified *Daqu* were mixed with steamed grains, and the corresponding fermented grains (FG) and liquors were obtained. Every FG sample (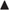) was collected from seven different positions (100 g FG at each position), and mixed well as one sample.

The grain fermentation (liquor brewing) was carried out according to the process shown in Figure 2A. Briefly, the steamed grains were cooled to below 40°C, mixed with 20% (based on the quantity of grain) of *Daqu*, then put into mud pits, and fermented for 2 months anaerobically. In the present experiment, the fermentation starters used were traditional, mixed, and fortified *Daqu*, respectively, and the mixed *Daqu* was composed of 50% traditional *Daqu* and 50% fortified *Daqu*. Therefore, the corresponding FG obtained after fermentation were referred to as FG0, FG50, and FG100, respectively.

### Sample Collection and Flavor Analysis

The samples were collected on 21 November 2017 according to the pattern shown in Figure 2B. For each mud pit, seven positions (100 g/each sample position) were taken from FG in the middle and bottom layer, respectively. These seven positions were mixed uniformly, taken 200 g as one sample, transferred to sterile polyethylene bags, and then stored at −20°C until further analysis. These samples were numbered as FG0-M, FG50-M, and FG100-M (middle layer samples), and FG0-B, FG50-B, and FG100-B (bottom layer samples), respectively. The corresponding fresh liquors distillated from these FG were also numbered L0-M, L50-M, L100-M, L0-B, L50-B, and L100-B, respectively. These six liquor samples were collected from the head, heart, and tail stages during distillation. For each liquor sample, five key points (100 mL/each point) were selected to sample at equal time intervals. About 500 mL was collected, pooled together, and immediately poured into glass bottles, then sealed and stored at 4°C until analysis. Extraction and determination of volatile compounds of the six liquor samples were done by headspace solid-phase microextraction (HS-SPME) and gas chromatography-mass spectrometry (GC-MS) according to the method described previously (Ding et al., 2017). Analysis of volatile compounds were conducted in triplicate and the results were expressed as mean ± standard deviation.

### DNA Extraction, PCR Amplification, and Sequence Analysis

Total bacterial genomic DNA samples were extracted using the Fast DNA SPIN extraction kits (MP Biomedicals, Santa Ana, CA, United States), following the manufacturer’s instructions, and stored at −20°C prior to further analysis. The quantity and quality of extracted DNA were measured using a NanoDrop ND-1000 spectrophotometer (Thermo Fisher Scientific, Waltham, MA, United States) and agarose gel electrophoresis, respectively. For bacteria, the V3-V4 domains of the 16S rRNA genes were amplified using primers 338F (5′-ACTCCTACGGGAGGCAGCA-3′) and 806R (5′-GGACTACHVGGGTWTCTAAT-3′). For fungi, the internal transcribed spacer ITS regions were amplified with primers ITS5 (5′-GGAAGTAAAAGTCGTAACAAGG-3′) and ITS1 (5′-GCTGCGTTCTTCATCGATGC-3′). Sample-specific 7-bp barcodes were incorporated into the primers for multiplex sequencing. The detailed PCR procedures were conducted according to a previous method (Li X. et al., 2014). PCR amplicons were purified with Agencourt AMPure Beads (Beckman Coulter, Indianapolis, IN) and quantified using the PicoGreen dsDNA Assay Kit (Invitrogen, Carlsbad, CA, United States). After the individual quantification step, amplicons were pooled in equal amounts, and pair-end 2 × 300 bp sequencing was performed using the Illumina MiSeq platform with MiSeq Reagent Kit v3 at Shanghai Personal Biotechnology Co., Ltd (Shanghai, China).

The Quantitative Insights Into Microbial Ecology (QIIME, v1.8.0) pipeline was employed to process the sequencing data as previously described (Caporaso et al., 2010). Briefly, raw sequencing reads with exact matches to the barcodes were assigned to respective samples and identified as valid sequences. The low-quality sequences (length below 150 bp, average Phred scores less than 20, mononucleotide repeats over 8 bp, and with ambiguous bases) were removed (Gill et al., 2006; Chen and Jiang, 2014). After chimera detection, the remaining high-quality sequences were clustered into operational taxonomic units (OTUs) at 97% sequence identity by UCLUST (Edgar, 2010). A representative sequence was selected from each OTU using default parameters. OTU taxonomic classification was conducted by BLAST searching the representative sequences set against the Greengenes Database (Desantis et al., 2006). An OTU table was further generated to record the abundance of each OTU in each sample and the taxonomy of these OTUs.

### Statistical Analysis

One-way analysis of variance (ANOVA) was carried out to evaluate significant differences (*p* < 0.05) in volatile compounds using SPSS 19.0 software (SPSS Inc. Chicago, IL, United States). Principal component analysis (PCA) was also conducted with SPSS 19.0 software. Sequence data analyses were mainly performed using QIIME (v1.8.0) and R packages (v3.2.0). OTU-level alpha diversity indices including Chao1 and ACE, were calculated using the OTU table in QIIME. Rarefaction curves were plotted via R packages. RDA between microbial community and volatile compounds was performed with Canoco 5.0 software.

## Results

### Richness and Diversity of Microbial Community

**FIGURE 3:**
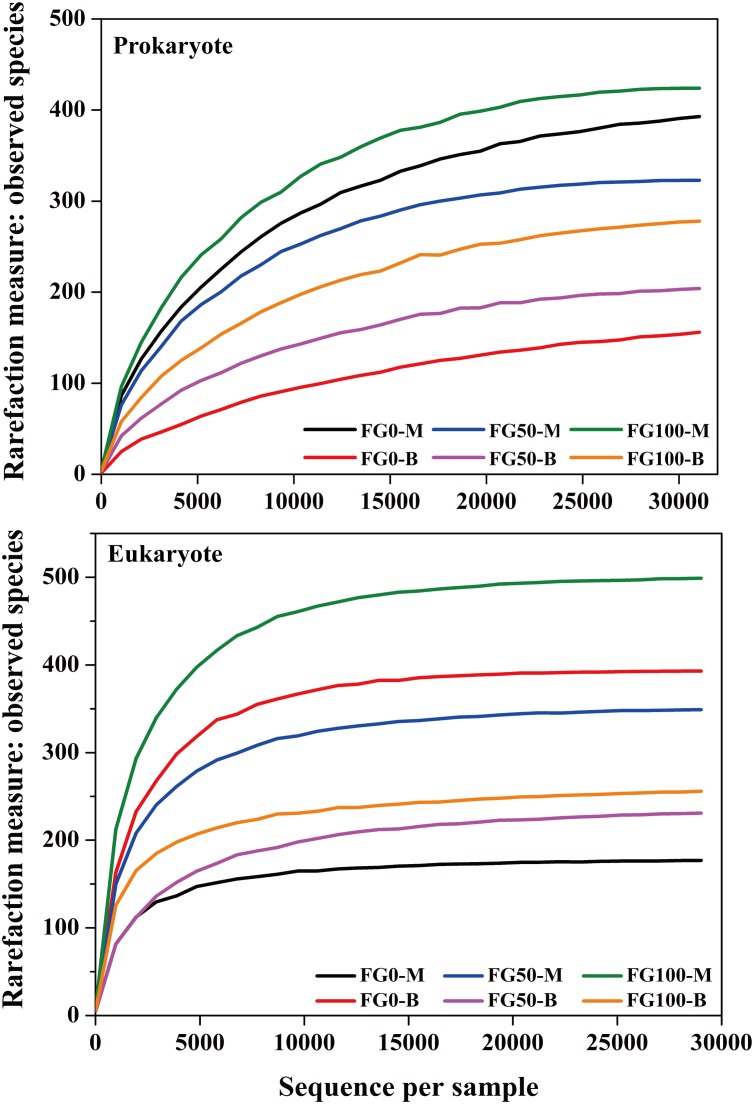
Rarefaction curve analysis for six FG samples fermented with different proportions of fortified *Daqu* based on the observed species.

An average of 31,060 and 29,020 effective tags in 16S rRNA and ITS sequences, respectively, were obtained after quality control from six samples. All the rarefaction curves based on the observed species were saturated (Figure 3), which indicated that the obtained sequenced reads were sufficient to represent the community diversity in these samples. The richness and diversity were characterized by Chao1 and Shannon, respectively, to describe the alpha-diversity of the microbial community in different samples (Table 1). For prokaryotes, the combined data showed higher microbial richness (Chao1) and diversity (Shannon) in the middle layer than that in the bottom layer from the same mud pit. It is worth noting that the richness and diversity of prokaryotes in the bottom layer increased when fortified *Daqu* was added. For eukaryotes, there were obvious differences in richness and diversity from different layers of FG. For FG0, Chao1 and Shannon in the bottom layer were higher than that in the middle layer, while in FG50 and FG100 the results were opposite. Interestingly, the richness and diversity of eukaryotes increased in the middle layer and decreased in the bottom layer when fortified *Daqu* was added.

**Table 1:** Richness and diversity indexes of microbial community in the six FG samples.

Sample	Prokaryote			Eukaryote		
	Chao1	Shannon	Observed species	Chao1	Shannon	Observed species
FG0-M	414	2.50	393	179	3.62	177
FG0-B	197	0.66	156	393	5.60	393
FG50-M	323	3.17	323	351	4.63	349
FG50-B	207	1.90	204	243	3.36	231
FG100-M	424	3.58	424	507	6.44	499
FG100-B	284	2.55	278	275	4.86	256

### Prokaryotic Microbial Community

A total of 7 phyla have been shared by all samples, including Firmicutes, Proteobacteria, Actinobacteria, Bacteroidetes, Acidobacteria, Chloroflexi, and Cyanobacteria (Supplementary Table S1). Among them, Firmicutes were the most abundant, which accounted for 91.4–98.6% of the total abundance, mainly including *Bacillus*, *Carnobacterium*, *Enterococcus*, *Lactobacillus*, *Lactococcus*, *Leuconostoc*, *Ruminococcus*, *Streptococcus*, *Thermo- actinomyces*, and *Virgibacillus* genera.

**FIGURE 4:**
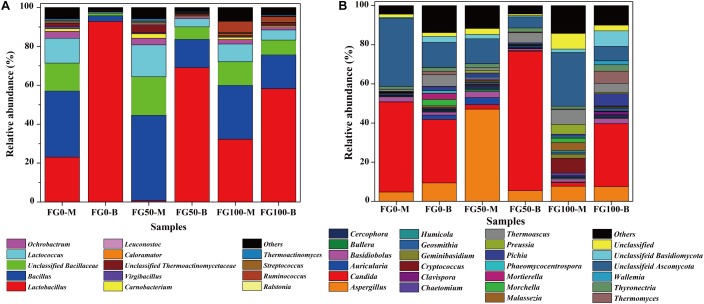
Relative abundance of prokaryotic **(A)** and eukaryotic **(B)** communities in FG at genus level.

Only genera that were detected in all mud pits were considered as an effective genus. A total of 95 genera were detected and clearly classified. The genera with a relative abundance over 0.5% in at least one sample were further analyzed to explore the effect of fortified *Daqu*, as shown in Figure 4A. There was a significant difference of community abundance in different layers of FG. For the middle layer (FG0-M, FG50-M, and FG100-M), *Bacillus* was the most dominant genus, while *Lactobacillus* was the most dominant genus in the bottom layer (FG0-B, FG50-B, and FG100-B). Specifically, the relative abundance of prokaryotes significantly changed in the bottom layer when fortified *Daqu* was used. Compared with FG0-B, the relative abundance of *Lactobacillus* dramatically decreased to 69.1 and 58.3% in FG50-B and FG100-B, respectively. Meanwhile FG50-B and FG100-B exhibited a higher relative abundance of the dominant genus *Bacillus* (14.6 and 17.4%) and 3 special genera including *Lactococcus* (4.2 and 5.2%), *Ochrobactrum* (1.1 and 1.6%), and *Carnobacterium* (0.52 and 0.68%) than that in FG0-B. In the middle layer, the relative abundance of *Bacillus*, *Bacillaceae*, *Lactococcus*, *Carnobacterium*, *Thermoactinomycetaceae*, *Leuconostoc*, *Streptococcus*, and *Thermoactinomyces* increased in FG50-M and decreased in FG100-M when compared with FG0-M. However, FG100 possessed a higher abundance of *Ruminococcus* (FG100-M 5.7 and FG100-B 2.5%) than that in FG0 and FG50.

### Eukaryotic Microbial Community

6 phyla were observed in the eukaryotic community, including Ascomycota, Zygomycota, Basidiomycota, Rozellomycota, Ciliophora, and Glomeromycota (Supplementary Table S2). The most abundant phylum was Ascomycota, with relative abundance from 70.9 to 95.8% in these samples, followed by Basidiomycota (1.8% ∼ 17.6%) and Zygomycota (1.3% ∼ 5.8%).

A total of 76 genera were identified and classified in these samples by the same data processing as prokaryotes. Among these genera, 22 abundant genera (with a relative abundance over 1% in at least one sample) affiliated with three phyla were observed (Figure 4B). Among them, 14 genera (*Aspergillus*, *Candida*, *Cercophora*, *Chaetomium*, *Clavispora*, *Geosmithia*, *Humicola*, *Morchella*, *Phaeomycocentrospora*, *Pichia*, *Preussia*, *Thermoascus*, *Thermomyces*, and *Thyronectria*), 6 genera (*Auricularia*, *Bullera*, *Cryptococcus*, *Geminibasidiu*, *Malassezia*, and *Wallemia*), and 2 genera (*Basidiobolus* and *Mortierella*) belonged to Ascomycota, Basidiomycota, and Zygomycota, respectively. The dominant genera *Aspergillus*, *Candida*, *Thermoascus* and other eukaryotic genera displayed a significant difference in these samples. Compared with FG0, FG50-M and FG50-B exhibited higher abundances of *Aspergillus* (47.1%) and *Candida* (71.3%), respectively. In the middle layer, more genera with a relative abundance over 1% in FG50-M and FG100-M were observed. In addition, the highest abundance of *Pichia* (6.3%) and *Thermomyces* (6.2%) were obtained in FG100-B.

### Analysis of Flavor Compound of Liquor

**FIGURE 5:**
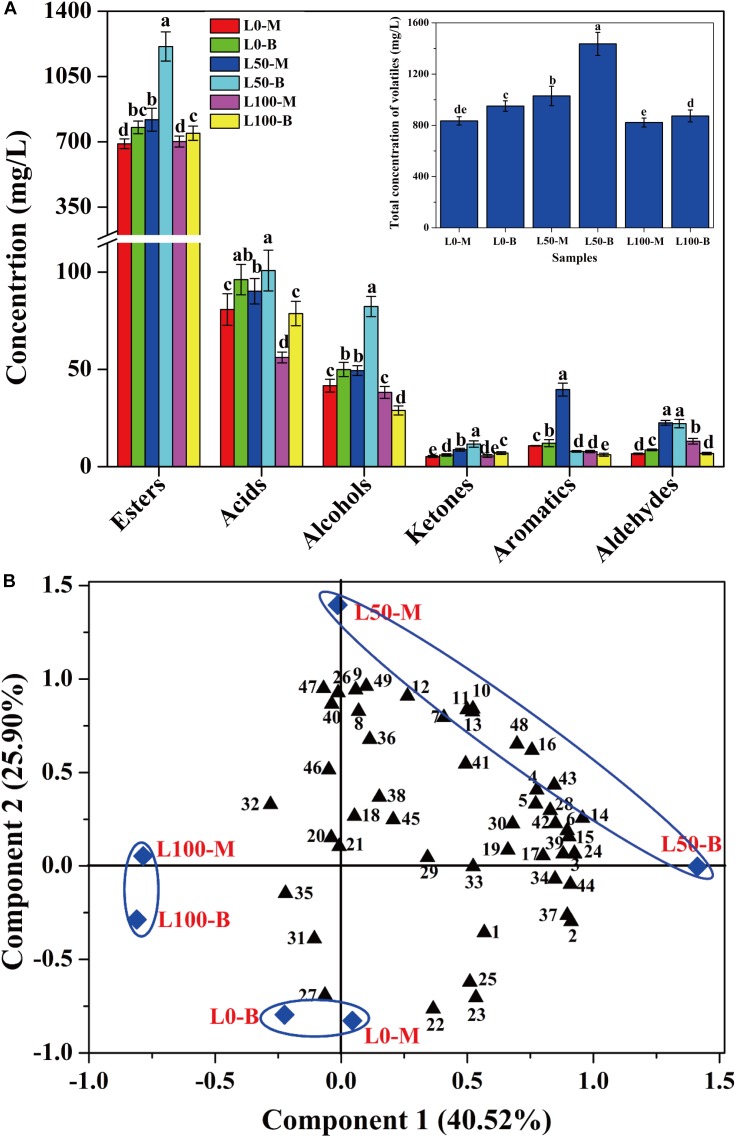
Concentration variation **(A)** and principal components analysis **(B)** of volatile compounds in liquors distillated from different FG. Different letters obtained by ANOVA indicate significant differences at *p* < 0.05 (*n* = 3). Each number in the atlas represents 1 compound, listed successively in Supplementary Table S3.

In order to assess the influences of fortified *Daqu* on the volatile profiles of fresh liquors, flavor compounds of liquors distillated from different FG were identified. A total of 49 flavor compounds including esters (30), acids (6), alcohols (4), ketones (4), aromatics (3), and aldehydes (2) were detected (Supplementary Table S3). Overall, significant differences of flavor compounds were observed in different liquors. As described in Figure 5A, compared with the liquor brewed without the addition of fortified *Daqu* (L0), a noteworthy increase in the total concentration of flavor compounds was observed in the L50 liquor (brewed with 50% fortified *Daqu* addition), reaching a maximum value of 1435.7 mg/L in L50-B. However, there was a slight variation in the total amount of flavor compounds between liquor L0 and liquor L100 (brewed with addition of 100% fortified *Daqu*). In terms of categories, the largest family of flavor compounds was esters, followed by acids, alcohols, and a small number of ketones, aromatics, and aldehydes. Likewise, the L50 liquor presented higher contents of these 6 flavor groups.

Further, the flavor compounds were used as variable vectors for chemometric analysis of samples to reveal the effect of fortified *Daqu* on liquor brewing. Two principal components (PC1 40.52 and PC2 25.90%) accounting for 66.42% of the total variance were extracted (Figure 5B). Interestingly, an obvious variation was observed by the PCA plot after the addition of fortified *Daqu*. Liquor L0 was mainly characterized by propyl hexanoate (No. 22), butyl hexanoate (No. 23), pentyl hexanoate (No. 25), and hexyl hexanoate (No. 27), while the flavor profile changed greatly by fortifying fermentation. Liquor L100 had higher Component 2 values than L0 and they were mainly characterized by acids including 3-methylbutanoic acid (No. 32), heptanoic acid (No. 35), and butanoic acid (No. 31). For liquor L50, L50-M and L50-B had higher Component 2 and Component 1 values, respectively, than L0, and they possessed higher contents of flavor compounds. L50-M was mainly characterized by the aromatic compounds including ethyl phenylacetate (No. 11), phenylethyl acetate (No. 12), benzeneethanol (No. 40), (2,2-diethoxyethyl)-benzene (No. 47), and benzaldehyde (No. 49). Meanwhile, L50-B was characterized by higher concentrations of important esters, including ethyl hexanoate (No. 1), ethyl heptanoate (No. 2), ethyl lactate (No. 3), ethyl octanoate (No. 4), ethyl hexadecanoate (No. 16), and isopentyl hexanoate (No. 24).

### Correlation Between Microbial Community and Flavor Compound

**FIGURE 6:**
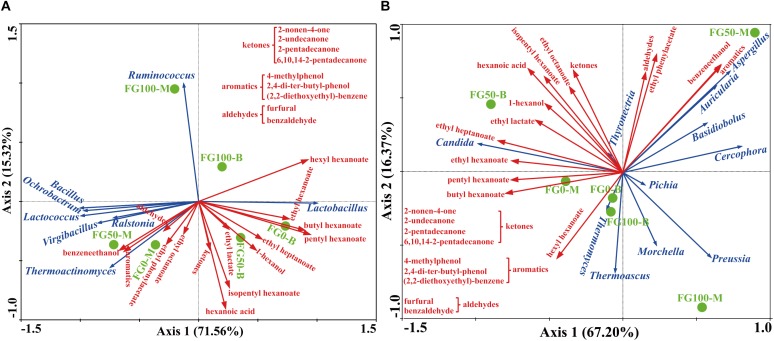
Correlation of prokaryotic **(A)** and eukaryotic **(B)** communities with flavor compounds by redundancy analysis.

In this study, RDA was conducted to reveal the possible relationship between microbial community and flavor compound, as shown in Figure 6. Overall, the two axes explained 86.88 and 83.57% of the variation in prokaryotic and eukaryotic community differentiation, respectively, suggesting the strong correlation between microbial community and flavor compounds. The hexanoic acid, 1-hexanol, and most esters, including ethyl hexanoate, butyl hexanoate, pentyl hexanoate, ethyl heptanoate, ethyl lactate, and isopentyl hexanoate were important compounds that strongly positively correlated with *Lactobacillus* in FG50-B. The aromatic compounds including benzaldehyde, 4-methylphenol, 2,4-di-ter-butyl-phenol, (2,2-diethoxyethyl)-benzene, benzeneethanol, and ethyl phenylacetate mainly correlated with community composition in FG50-M with abundant *Bacillus*, *Ochrobactrum*, *Lactococcus*, *Virgibacillus*, *Ralstonia*, and *Thermoactinomyces*. In particular, *Ruminococcus* was only positively correlated with hexyl hexanoate. For eukaryotes, ethyl esters (ethyl hexanoate, ethyl heptanoate, and ethyl lactate) and 1-hexanol were strongly positively correlated with *Candida* in FG50-B. *Aspergillus*, *Auricularia*, *Basidiobolus*, and *Cercophora* were positively correlated with aldehydes, aromatics, benzeneethanol, and ethyl phenylacetate. In addition, hexyl hexanoate was mainly positively correlated with a community composition in FG100-B with higher abundance of *Thermomyces* and *Thermoascus*.

## Discussion

The brewing process of Chinese strong-flavor liquor is complicated and involves many microbes originating from *Daqu* and pit mud. Especially for *Daqu*, the contribution of microbes to fermented grain is very important. For example, Wang X. et al. (2017) found that *Daqu* was the main source of strict aerobes and facultative aerobes, and they took up over 74% of prokaryotic communities in fermented grain. To stabilize and improve the quality of Chinese liquor, the corresponding strategies of both fortified *Daqu* manufacture and artificial pit mud culture were developed. Artificial pit mud is indeed applied widely in Chinese liquor production (Ding et al., 2014; Zhang L. et al., 2015; Sun et al., 2017). Unfortunately, artificial pit mud is generally continuously used and easily aged during the fermentation process. An obvious characteristic of aged artificial pit mud is the formation of a large amount of ferrous lactate, magnesium lactate, and copper lactate (Zhang Y. et al., 2014; Sun et al., 2017), and this leads to fermentation failure.

In recent years, interest in fortified *daqu* for liquor making has increased in many researchers. For example, highly similar dynamics of physicochemical parameters, enzymatic activities, and microbial communities were observed during the traditional and fortified *Daqu* fermentation processes (Li P. et al., 2017). Another study has shown that inoculation of *Bacillus licheniformis* influenced the enzyme activity of *Daqu*, including a significant increase in amylase activity, and the significant decrease in glucoamylase activity and esterase activity (Wang P. et al., 2017). Moreover, liquor yield, liquor flavor, and sensory appraisals were also compared by applying fortified *Daqu* and traditional *Daqu* for liquor production (Wu et al., 2009). Taken together, these studies only focused on the manufacture of fortified *Daqu* by evaluating physicochemical properties, enzyme activities, and microbial community structures, as well as by applying analyses of percent conversion and flavor characteristics to liquor production. Nevertheless, little attention has been paid to the bioturbation effects of fortified *Daqu* (especially for functional microbiota) on the community structure of the brewing process and their metabolisms. This study aimed to investigate the complex microbial community in the FG with different proportions of fortified *Daqu* used and to evaluate the effect of functional microbiota on the flavor of strong-flavor liquor.

In this study, the microbial communities were mainly dominated by three prokaryotic genera (*Bacillus*, *Lactobacillus*, and *Lactococcus*) and three eukaryotic genera (*Aspergillus*, *Candida*, and *Thermoascus*), which is consistent with many previous reports (Sun et al., 2016; Li H. et al., 2017; Wang X. et al., 2017). Clear differences of community compositions in different layers of FG were observed (Table 1 and Figure 4). The heterogeneity of microbial communities in different layers might be an important reason why liquors obtained from different layers exhibited different flavor compounds. For prokaryotes, *Lactobacillus* seemed to prefer to inhabit the bottom layer because of the anaerobic environments. A higher abundance of *Bacillus* (27.7% ∼ 44.0%) in the middle layer was observed possibly because the oxygen content is higher than that in the bottom layer. Eukaryotic diversity was lower in the middle layer by some aerobic eukaryotes with the disadvantage of competition for growth with the abundant *Bacillus* in FG0. In FG50 and FG100, acid production by abundant *Lactobacillus* might have inhibited other eukaryotes, resulting in a decrease of eukaryotic diversity in the bottom layer. Thus, as a disturbance, the fortified *Daqu* could regulate the microbial structure in the brewing ecosystem.

When fermented with fortified *Daqu*, as expected, the total relative abundance of *Bacillus* (sum of the proportion of the middle layer and the proportion of the bottom layer) increased in FG50 and FG100, and the highest abundance of *Bacillus* was observed in FG50-M (Figure 4A). *Bacillus* could secret various hydrolases including amylases, proteases, and lipases for macromolecular hydrolysis and produce flavor compounds in the brewing process (Yan et al., 2013b; Li H. et al., 2014; Li Z. et al., 2014). For example, *Bacillus licheniformis* could produce aromatic compounds, C4 compounds, pyrazines, and volatile acids by solid-state fermentation (Zhang et al., 2013). More importantly, improvement of hydrolase and flavor precursor production was obtained when *Bacillus* was co-cultured (Hero et al., 2017; Samad and Zainol, 2017). The corresponding liquor L50-M also exhibited higher contents of aromatic compounds, including 2,4-di-ter-butyl-phenol, (2,2-diethoxyethyl)-benzene, benzeneethanol, and ethyl phenylacetate (Supplementary Table S3). Especially for benzeneethanol and ethyl phenylacetate, they could endow rose-like and honey fragrances to the liquor (Fan and Qian, 2006a). Furthermore, RDA results indicated that these aromatic compounds were positively correlated with *Bacillus* in FG50-M (Figure 6A). Likewise, the relative abundance of *Aspergillus* increased to the highest concentration (47.1%) in FG50-M (Figure 4B), and *Aspergillus* showed a positive correlation with these aromatic compounds (Figure 6B). *Aspergillus* produce a wide spectrum of hydrolase for starch saccharification, protein hydrolysis, and flavonoid formation (Machida et al., 2008). Overall, *Bacillus* and *Aspergillus* were positively correlated with aromatic compounds, which was in agreement with the notion that *Aspergillus* produced higher contents of aromatic phenols, including 2-methoxyphenol, 2-methoxy-4-vinylphenol, and 4-ethyl-2-methoxyphenol in co-culture with *Bacillus licheniformis* (Wang P. et al., 2017). But this is slightly different from our results, because in addition to the aromatic phenols, the aromatic compounds also included ethyl phenylacetate, benzaldehyde, furfural, and benzeneethanol in our study. Therefore, when fermented with 50% fortified *Daqu*, the increased relative abundance of *Bacillus* and *Aspergillus* in FG may accelerate the brewing process and produce more aromatic compounds.

As for *Lactobacillus*, it could produce lactic acid from glucose, and the lactic acid can be further converted by enzymatic reaction into ethyl lactate, which can improve the mellow flavor of Chinese liquor (Gao et al., 2014). However, compared with FG fermented without the addition of fortified *Daqu*, the abundance of *Lactobacillus* significantly decreased in FG50-B (Figure 4A). Interestingly, the content of ethyl lactate was the highest (128.01 mg/L) in the corresponding liquor L50-B (Supplementary Table S3), and this could be attributed to the fact that (i) the relative abundance of *Candida* increased to the highest (71.3%) in FG50-B, (ii) the other lactic acid bacteria *Lactococcus*, *Leuconostoc*, and *Streptococcus* also can produce lactic acid, and (iii) lactic acid, together with ethanol produced by *Candida*, gets esterified into ethyl lactate. Additionally, L50-B presented higher levels of other important esters, including ethyl hexanoate, ethyl heptanoate, ethyl octanoate, butyl hexanoate, isopentyl hexanoate, and pentyl hexanoate (Supplementary Table S3). These esters also play pivotal roles in contributing fruity and floral flavors for Chinese liquor (Fan and Qian, 2006b). Moreover, RDA results also showed that *Lactobacillus* and *Candida* were highly positively correlated with these esters (Figure 6). These results indicated that *Lactobacillus*, together with *Candida*, can improve ester production through synergistic interactions and enhance the fruity flavor of liquor when brewed with 50% fortified *Daqu*.

Together, variations of microbial community with RDA results suggested that *Lactobacillus* and other prokaryotic genera were highly competitive during the brewing process. It could be confirmed by the correlations between *Lactobacillus* and genera *Bacillus*, *Ruminococcus*, *Thermoactinomyces*, *Lactococcus*, *Ochrobactrum*, *Ralstonia*, and *Virgibacillus* (Figure 6A). A negative correlation between *Lactobacillus* and the genera observed in this study is in agreement with that confirmed from the previously studied fermented grain (Wang X. et al., 2017). In addition, the abundance of *Lactobacillus* was found to be negatively correlated with hexanoic acid production as previously reported (Tao et al., 2014), and the liquor L50-B presented the highest content (73.02 mg/L) of hexanoic acid in the present study (Supplementary Table S3). These results demonstrated that an appropriate abundance of *Lactobacillus* and the exclusion relationship with other genera could help to produce hexanoic acid and maintain the stability of fermentation.

In particular, hexyl hexanoate was positively correlated with highly abundant *Ruminococcus* in the FG100 (Figure 6A). *Ruminococcus* was identified as the most dominant member of *Clostridiales*, showing a good ability for starch degradation (Mukhopadhya et al., 2017). Additionally, hydrogen produced by *Ruminococcus* could be transferred to *Vibrio succinogenes*, which was contributive to growth of *Vibrio succinogenes* (Iannotti et al., 1973). The main mechanism of synergistic interactions is interspecies hydrogen transfer in the brewing process (Thauer et al., 2008), and this mechanism helps to produce hexanoic acid by hexanoic acid bacteria (Tao et al., 2017). In this study, the important thermophilic fungi *Thermoascus* and *Thermomyces* possessed high abundance just below *Aspergillus* and *Candida* in the FG100 (Figure 4B), and they were also positively correlated with hexyl hexanoate (Figure 6B). *Thermoascus* and *Thermomyces* have all been reported to be high producers of thermophilic enzymes for carbohydrate degradation (Nguyen et al., 2002; Mcclendon et al., 2012). Taken together, *Ruminococcus*, *Thermoascus*, and *Thermomyces* could be the core genera in the brewing process and related to the formation of hexyl hexanoate.

**FIGURE 7:**
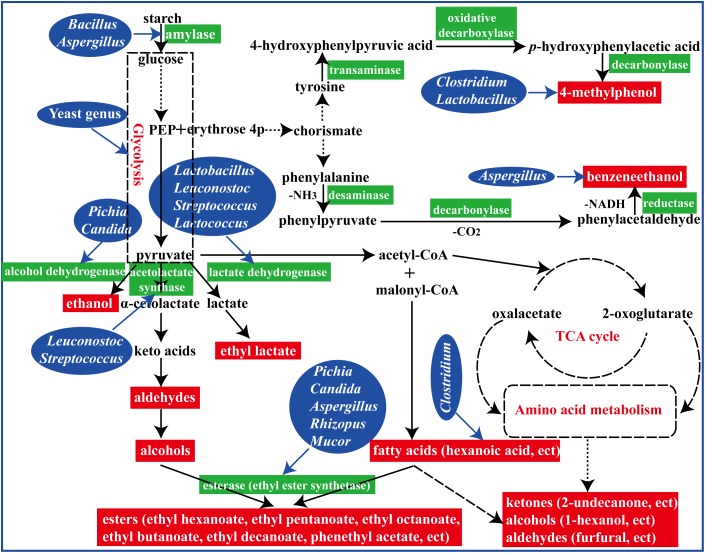
A schematic representation of formation of flavor compounds by microbial metabolism in the brewing process.

In summary, fermented with an appropriate proportion of fortified *Daqu* (manufactured by inoculation *Bacillus velezensis* and *Bacillus subtilis*) evoked a significant regulation in microbial community structure and their metabolisms in the brewing process (Figure 7). To reveal the perturbation mechanism of *Bacillus* on brewing process, the effect of *Bacillus* on the microbial community and formation of volatile compounds in brewing process has been investigated. *Bacillus* could produce amylase for starch degradation, which could help to accelerate the brewing process. In addition, the microbial community structure in the FG was changed after inoculation of *Bacillus* by synergistic or exclusive interactions between the microbes. Furthermore, higher contents of aromatic compounds and main esters were observed in the liquor fermented with 50% fortified *Daqu*, which were mainly correlated with *Bacillus*, *Aspergillus*, *Lactobacillus*, and *Candida*. The results presented in this study indicated that *Bacillus*, as an exogenous disturbance, regulated the microbial community structure of the brewing ecosystem and played its own role.

## Author Contributions

GH performed the experiments, analyzed the data, and prepared the manuscript. JH performed the experiments and contributed to manuscript discussion. RZ contributed to the experimental design, manuscript revision, and overall support of this study. CW and YJ contributed to manuscript revision.

## Conflict of Interest Statement

The authors declare that the research was conducted in the absence of any commercial or financial relationships that could be construed as a potential conflict of interest.

## References

[B1] Caporaso J. G. Kuczynski J. Stombaugh J. Bittinger K. Bushman F. D. Costello E. K. (2010). QIIME allows analysis of high-throughput community sequencing data. *Nat. Methods* 7 335–336. 10.1038/nmeth.f.303 20383131 PMC3156573

[B2] Chen H. Jiang W. (2014). Application of high-throughput sequencing in understanding human oral microbiome related with health and disease. *Front. Microbiol.* 5:508. 10.3389/fmicb.2014.00508 25352835 PMC4195358

[B3] Desantis T. Z. Hugenholtz P. Larsen N. Rojas M. Brodie E. L. Keller K. (2006). Greengenes, a chimera-checked 16S rRNA gene database and workbench compatible with ARB. *Appl. Environ. Microbiol.* 72 5069–5072. 10.1128/AEM.03006-05 16820507 PMC1489311

[B4] Ding X. Huang J. Wu C. Zhou R. (2017). Effects of different distillation patterns on main compounds of Chinese Luzhou-flavour raw liquors. *J. Inst. Brew.* 123 442–451. 10.1002/jib.422

[B5] Ding X. Wu C. Huang J. Li H. Zhou R. (2014). Eubacterial and archaeal community characteristics in the man-made pit mud revealed by combined PCR-DGGE and FISH analyses. *Food Res. Int.* 62 1047–1053. 10.1016/j.foodres.2014.05.045

[B6] Edgar R. C. (2010). Search and clustering orders of magnitude faster than BLAST. *Bioinformatics* 26 2460–2461. 10.1093/bioinformatics/btq461 20709691

[B7] Fan W. Qian M. C. (2006a). Characterization of aroma compounds of Chinese “Wuliangye” and “Jiannanchun” liquors by aroma extract dilution analysis. *J. Agric. Food Chem.* 54 2695–2704. 10.1021/jf052635t 16569063

[B8] Fan W. Qian M. C. (2006b). Identification of aroma compounds in Chinese “Yanghe Daqu” liquor by normal phase chromatography fractionation followed by gas chromatography [sol] olfactometry. *Flavour Fragr. J.* 21 333–342. 10.1002/ffj.1621

[B9] Gao W. Fan W. Xu Y. (2014). Characterization of the key odorants in light aroma type chinese liquor by gas chromatography-olfactometry, quantitative measurements, aroma recombination, and omission studies. *J. Agric. Food Chem.* 62 5796–5804. 10.1021/jf501214c 24909925

[B10] Gill S. R. Pop M. Deboy R. T. Eckburg P. B. Turnbaugh P. J. Samuel B. S. (2006). Metagenomic analysis of the human distal gut microbiome. *Science* 312 1355–1359. 10.1126/science.1124234 16741115 PMC3027896

[B11] Gou M. Wang H. Z. Yuan H. W. Zhang W. X. Tang Y. Q. Kida K. (2015). Characterization of the microbial community in three types of fermentation starters used for Chinese liquor production. *J. Inst. Brew.* 121 620–627. 10.1002/jib.272

[B12] Guo J. Sun L. Guo H. Zou D. Liu X. Wang Y. (2018). Effect of cellulase-producing bacteria on fungi community structure and ester generation in Chinese liquor fermenting grains. *J. Am. Soc. Brew. Chem.* 76 130–140. 10.1080/03610470.2018.1424401

[B13] Guo J. H. Sun L. H. Liu X. L. (2017). Effects of cellulase-producing bacteria on bacterial community structure and diversity during fermentation of Chinese liquor grains. *J. Inst. Brew.* 123 130–137. 10.1002/jib.390

[B14] Hero J. S. Pisa J. H. Perotti N. I. Romero C. M. Martínez M. A. (2017). Endoglucanase and xylanase production by *Bacillus* sp. AR03 in co-culture. *Prep. Biochem. Biotechnol.* 47 589–596. 10.1080/10826068.2017.1280826 28106512

[B15] Huang Y. Yi Z. Jin Y. Zhao Y. He K. Liu D. (2017). New microbial resource: microbial diversity, function and dynamics in Chinese liquor starter. *Sci. Rep.* 7:14577. 10.1038/s41598-017-14968-8 29109406 PMC5674051

[B16] Iannotti E. L. Kafkewitz D. Wolin M. J. Bryant M. P. (1973). Glucose fermentation products in *Ruminococcus albus* grown in continuous culture with *Vibrio succinogenes*: changes caused by interspecies transfer of H2. *J. Bacteriol.* 114 1231–1240. 4351387 10.1128/jb.114.3.1231-1240.1973 PMC285387

[B17] Jin G. Zhu Y. Xu Y. (2017). Mystery behind Chinese liquor fermentation. *Trends Food Sci. Technol.* 63 18–28. 10.1016/j.tifs.2017.02.016

[B18] Li H. Huang J. Liu X. Zhou R. Ding X. Xiang Q. (2017). Characterization of interphase microbial community in Luzhou-flavored liquor manufacturing pits of various ages by polyphasic detection methods. *J. Microbiol. Biotechnol.* 27 130–140. 10.4014/jmb.1605.05036 27713211

[B19] Li P. Lin W. Liu X. Wang X. Gan X. Luo L. (2017). Effect of bioaugmented inoculation on microbiota dynamics during solid-state fermentation of Daqu starter using autochthonous of *Bacillus*, *Pediococcus*, *Wickerhamomyces* and *Saccharomycopsis*. *Food Microbiol.* 61 83–92. 10.1016/j.fm.2016.09.004 27697173

[B20] Li H. Lian B. Ding Y. Nie C. Zhang Q. (2014). Bacterial diversity in the central black component of Maotai Daqu and its flavor analysis. *Ann. Microbiol.* 64 1659–1669. 10.1007/s13213-014-0809-z

[B21] Li X. Rui J. Mao Y. Yannarell A. Mackie R. (2014). Dynamics of the bacterial community structure in the rhizosphere of a maize cultivar. *Soil Biol. Biochem.* 68 392–401. 10.1016/j.soilbio.2013.10.017

[B22] Li Z. Bai Z. Wang D. Zhang W. Zhang M. Lin F. (2014). Cultivable bacterial diversity and amylase production in three typical Daqus of Chinese spirits. *Int. J. Food Sci. Technol.* 49 776–786. 10.1111/ijfs.12365

[B23] Machida M. Yamada O. Gomi K. (2008). Genomics of *Aspergillus oryzae*: learning from the history of koji mold and exploration of its future. *DNA Res.* 15 173–183. 10.1093/dnares/dsn020 18820080 PMC2575883

[B24] Mcclendon S. D. Batth T. Petzold C. J. Adams P. D. Simmons B. A. Singer S. W. (2012). *Thermoascus aurantiacus* is a promising source of enzymes for biomass deconstruction under thermophilic conditions. *Biotechnol. Biofuels* 5:54. 10.1186/1754-6834-5-54 22839529 PMC3507748

[B25] Mukhopadhya I. Morais S. Laverde-Gomez J. Sheridan P. O. Walker A. W. Kelly W. (2017). Sporulation capability and amylosome conservation among diverse human colonic and rumen isolates of the keystone starch-degrader *Ruminococcus bromii*. *Environ. Microbiol.* 20 324–336. 10.1111/1462-2920.14000 29159997 PMC5814915

[B26] Nguyen Q. D. Rezessy-Szabó J. M. Claeyssens M. Stals I. Hoschke Á. (2002). Purification and characterisation of amylolytic enzymes from thermophilic fungus *Thermomyces lanuginosus* strain ATCC 34626. *Enzyme Microb. Technol.* 31 345–352. 10.1016/S0141-0229(02)00128-X

[B27] Pan L. Lin W. Xiong L. Wang X. Luo L. (2016). Environmental factors affecting microbiota dynamics during traditional solid-state fermentation of Chinese Daqu starter. *Front. Microbiol.* 7:1237. 10.3389/fmicb.2016.01237 27540378 PMC4972817

[B28] Samad K. A. Zainol N. (2017). Effects of agitation and volume of inoculum on ferulic acid production by co-culture. *Biocatal. Agric. Biotechnol.* 10 9–12. 10.1016/j.bcab.2017.01.010

[B29] Sun W. Xiao H. Peng Q. Zhang Q. Li X. Han Y. (2016). Analysis of bacterial diversity of Chinese Luzhou-flavor liquor brewed in different seasons by Illumina Miseq sequencing. *Ann. Microbiol.* 66 1293–1301. 10.1007/s13213-016-1223-5

[B30] Sun Z. Chen C. Hou X. Zhang J. Tian F. Li C. (2017). Prokaryotic diversity and biochemical properties in aging artificial pit mud used for the production of Chinese strong flavor liquor. *3 Biotech* 7:335. 10.1007/s13205-017-0978-0 28955632 PMC5605472

[B31] Tao Y. Li J. Rui J. Xu Z. Zhou Y. Hu X. (2014). Prokaryotic communities in pit mud from different-aged cellars used for the production of Chinese strong-flavored liquor. *Appl. Environ. Microbiol.* 80 2254–2260. 10.1128/AEM.04070-13 24487528 PMC3993157

[B32] Tao Y. Wang X. Li X. Wei N. Jin H. Xu Z. (2017). The functional potential and active populations of the pit mud microbiome for the production of Chinese strong-flavour liquor. *Microbial Biotechnol.* 10 1603–1615. 10.1111/1751-7915.12729 28703874 PMC5658580

[B33] Thauer R. Kaster A. Seedorf H. W. Hedderich R. (2008). Methanogenic archaea: ecologically relevant differences in energy conservation. *Nat. Rev. Microbiol.* 6 579–591. 10.1038/nrmicro1931 18587410

[B34] Wang P. Wu Q. Jiang X. Wang Z. Tang J. Xu Y. (2017). *Bacillus licheniformis* affects the microbial community and metabolic profile in the spontaneous fermentation of Daqu starter for Chinese liquor making. *Int. J. Food Microbiol.* 250 59–67. 10.1016/j.ijfoodmicro.2017.03.010 28371716

[B35] Wang X. Du H. Xu Y. (2017). Source tracking of prokaryotic communities in fermented grain of Chinese strong-flavor liquor. *Int. J. Food Microbiol.* 244 27–35. 10.1016/j.ijfoodmicro.2016.12.018 28064120

[B36] Wu Z. Zhang W. X. Zhang Q. S. Hu C. Wang R. Liu Z. H. (2009). Developing new sacchariferous starters for liquor production based on functional strains isolated from the pits of several famous Luzhou-flavor liquor brewers. *J. Inst. Brew.* 115 111–115. 10.1002/j.2050-0416.2009.tb00354.x

[B37] Xiao C. Lu Z. M. Zhang X. J. Wang S. T. Ao L. Shen C. H. (2017). Bio-heat is a key environmental driver shaping microbial community of medium-temperature Daqu. *Appl. Environ. Microbiol.* 83:AEM.1550–AEM.1517. 10.1128/AEM.01550-17 28970223 PMC5691423

[B38] Xu Y. Sun B. Fan G. Teng C. Xiong K. Zhu Y. (2017). The brewing process and microbial diversity of strong flavour Chinese spirits: a review. *J. Inst. Brew.* 123 5–12. 10.1002/jib.404

[B39] Yan Z. Zheng X. W. Chen J. Y. Han J. S. Han B. Z. (2013a). Effect of different *Bacillus* strains on the profile of organic acids in a liquid culture of Daqu. *J. Inst. Brew.* 119 78–83. 10.1002/jib.58

[B40] Yan Z. Zheng X. W. Han B. Z. Han J. S. Nout M. J. Chen J. Y. (2013b). Monitoring the ecology of *Bacillus* during Daqu incubation, a fermentation starter, using culture-dependent and culture-independent methods. *J. Microbiol. Biotechnol.* 23 614–622. 10.4014/jmb.1211.11065 23648849

[B41] Zhang L. Wu C. Ding X. Zheng J. Zhou R. (2014). Characterisation of microbial communities in Chinese liquor fermentation starters Daqu using nested PCR-DGGE. *World J. Microb. Biotechnol.* 30 3055–3063. 10.1007/s11274-014-1732-y 25193747

[B42] Zhang Y. Cong J. Lu H. Yang C. Yang Y. Zhou J. (2014). An integrated study to analyze soil microbial community structure and metabolic potential in two forest types. *PLoS One* 9:e93773. 10.1371/journal.pone.0093773 24743581 PMC3990527

[B43] Zhang L. Zhou R. Niu M. Zheng J. Wu C. (2015). Difference of microbial community stressed in artificial pit muds for Luzhou-flavour liquor brewing revealed by multiphase culture-independent technology. *J. Appl. Microbiol.* 119 1345–1356. 10.1111/jam.12943 26303819

[B44] Zhang R. Wu Q. Xu Y. (2013). Aroma characteristics of Moutai-flavour liquor produced with *Bacillus licheniformis* by solid-state fermentation. *Lett. Appl. Microbiol.* 57 11–18. 10.1111/lam.12087 23594087

[B45] Zheng X. W. Han B. Z. (2016). Baijiu, Chinese liquor: history, classification and manufacture. *J. Ethnic Foods.* 3 19–25. 10.1016/j.jef.2016.03.001

[B46] Zheng X. W. Tabrizi M. R. Nout M. J. R. Han B. Z. (2012). Daqu: a traditional Chinese liquor fermentation starter. *J. Inst. Brew.* 117 82–90. 10.1002/j.2050-0416.2011.tb00447.x

[B47] Zheng X. W. Yan Z. Nout M. J. Boekhout T. Han B. Z. Zwietering M. H. (2015). Characterization of the microbial community in different types of Daqu samples as revealed by 16S rRNA and 26S rRNA gene clone libraries. *World J. Microb. Biotechnol.* 31 199–208. 10.1007/s11274-014-1776-z 25395233

[B48] Zou W. Ye G. Zhang K. (2018). Diversity, function, and application of *Clostridium* in Chinese strong flavor baijiu ecosystem: a review. *J. Food Sci.* 83 1193–1199. 10.1111/1750-3841.14134 29660763

