# Supplementary material for: Effect of Fortified Daqu on the Microbial Community and Flavor in Chinese Strong-Flavor Liquor Brewing Process
Source: Front Microbiol. 2019 Jan 29;10:56. doi: 10.3389/fmicb.2019.00056 (PMC6361764; doi:10.3389/fmicb.2019.00056)
Supplement: Supplementary file 1 [file Table_1.docx]

**Effect of fortified *Daqu* on the microbial community and flavor in Chinese strong-flavor liquor brewing process**

Guiqiang He^1,2^, Jun Huang^1,2^, Rongqing Zhou^1,2,3*^, Suyi Zhang^3^, Yi Dong^3^, Chongde Wu^1,2^, Yao Jin^1,2^, Xiaojun Wang^3^

^1^ College of Light Industry, Textile & Food Engineering, Sichuan University, Chengdu 610065, China;

^2^ Key Laboratory of Leather Chemistry and Engineering, Ministry of Education, Sichuan University, Chengdu 610065, China;

^3^ National Engineering Research Center of Solid-State Manufacturing, Luzhou 646000, China.

***Corresponding author:**

Rongqing Zhou

Mailing address: College of Light Industry, Textile & Food Engineering, Sichuan University, Chengdu 610065, China.

Phone: +86-28-85406149, Fax: +86-28-85405237

E-mail: [zhourqing@scu.edu.cn](mailto:zhourqing@scu.edu.cn)

**Table S1** Relative abundance of prokaryotic community in FG at the phylum level.

| Phyla | Relative abundance (%) | | | | | |
| --- | --- | --- | --- | --- | --- | --- |
|  | FG0-M | FG0-B | FG50-M | FG50-B | FG100-M | FG100-B |
| *Acidobacteria* | 0.019 | 0.011 | 0.017 | 0.005 | 0.047 | 0.007 |
| *Actinobacteria* | 0.668 | 0.333 | 0.676 | 0.255 | 1.023 | 0.321 |
| *Bacteroidetes* | 0.473 | 0.067 | 0.584 | 0.127 | 0.813 | 0.627 |
| *Chloroflexi* | 0.025 | 0.020 | 0.081 | 0.032 | 0.087 | 0.027 |
| *Cyanobacteria* | 0.059 | 0.007 | 0.035 | 0.005 | 0.089 | 0.014 |
| *Firmicutes* | 91.397 | 98.587 | 92.065 | 97.352 | 92.937 | 95.678 |
| *Proteobacteria* | 7.173 | 0.909 | 6.410 | 2.165 | 4.706 | 3.230 |

**Table S2** Relative abundance of eukaryotic community in FG at the phylum level.

| Phyla | Relative abundance (%) | | | | | |
| --- | --- | --- | --- | --- | --- | --- |
|  | FG0-M | FG0-B | FG50-M | FG50-B | FG100-M | FG100-B |
| *Ascomycota* | 92.567 | 81.378 | 81.956 | 95.799 | 70.880 | 78.915 |
| *Basidiomycota* | 2.147 | 9.556 | 9.453 | 1.790 | 17.607 | 13.310 |
| *Ciliophora* | 0.257 | 0.029 | 0.317 | 0.033 | 0.409 | 0.573 |
| *Glomeromycota* | 0.086 | 0.220 | 0.136 | 0.013 | 0.107 | 0.098 |
| *Rozellomycota* | 0.545 | 1.034 | 0.494 | 0.184 | 0.588 | 0.854 |
| *Zygomycota* | 2.816 | 5.753 | 4.473 | 1.287 | 2.128 | 3.135 |
| *Unclassified* | 1.582 | 2.031 | 3.171 | 0.894 | 8.281 | 3.115 |

**Table S3** Concentration of volatile compounds in liquors distillated from different FG.

| Number Compounds | | Concentration (mg/L) | | | | | |
| --- | --- | --- | --- | --- | --- | --- | --- |
|  |  | Addition 0% fortified Daqu | | Addition 50% fortified Daqu | | Addition 0% fortified Daqu | |
|  |  | L0-M | L0-B | L50-M | L50-B | L100-M | L100-B |
| **Esters (30)** | | | | | | | |
| 1 | ethyl hexanoate | 175.02±7.56^d^ | 211.30±7.49^c^ | 166.66±9.81^d^ | 254.26±19.38^a^ | 173.82±6.78^d^ | 229.68±5.48^b^ |
| 2 | ethyl heptanoate | 26.48±3.88^b^ | 28.51±1.97^b^ | 18.31±4.12^c^ | 51.82±2.16^a^ | 22.11±1.21^c^ | 24.25±1.32^c^ |
| 3 | ethyl lactate | 20.39±2.57^c^ | 21.40±0.04^c^ | 29.70±3.88^b^ | 128.01±4.86^a^ | 32.42±1.73^b^ | 15.78±1.59^d^ |
| 4 | ethyl octanoate | 75.21±1.81^d^ | 55.64±4.40^e^ | 96.99±2.15^b^ | 130.20±13.07^a^ | 74.75±3.94^d^ | 85.32±3.90^c^ |
| 5 | ethyl nonanoate | 8.99±0.19^c^ | 6.68±0.16^d^ | 10.43±0.05^b^ | 14.85±0.07^a^ | 10.93±0.53^b^ | 6.79±1.52^cd^ |
| 6 | ethyl 2-hydroxy-4-methylvalerate | 9.69±1.69^d^ | 15.35±3.05^c^ | 24.59±3.37^b^ | 70.59±6.22^a^ | 22.17±1.12^b^ | 10.60±0.30^d^ |
| 7 | ethyl decanoate | 17.93±0.49^c^ | 9.51±0.13^d^ | 30.33±3.25^a^ | 24.61±2.55^b^ | 21.17±1.53^b^ | 10.39±2.34^d^ |
| 8 | ethyl benzoate | 1.56±0.02^d^ | 1.80±0.09^d^ | 4.60±0.28^a^ | 3.55±0.36^b^ | 4.33±0.25^a^ | 2.43±0.04^c^ |
| 9 | diethyl succinate | 0.51±0.10^d^ | 0.16±0.02^e^ | 2.25±0.05^a^ | 0.69±0.03^c^ | 0.88±0.05^b^ | 0.10±0.03^e^ |
| 10 | ethyl trans-2-decenoate | 0.34±0.04^b^ | 0.17±0.00^d^ | 0.93±0.01^a^ | 0.74±0.24^a^ | 0.35±0.07^b^ | 0.27±0.10^c^ |
| 11 | ethyl phenylacetate | 18.93±1.36^d^ | 26.37±0.72^c^ | 51.11±2.34^a^ | 44.29±0.43^b^ | 26.62±1.59^c^ | 24.93±0.83^c^ |
| 12 | phenylethyl acetate | 1.70±0.00^c^ | 1.51±0.11^c^ | 5.90±0.64^a^ | 2.85±0.03^b^ | 1.63±0.28^c^ | 0.85±0.25^d^ |
| 13 | ethyl 3-phenylpropanoate | 7.66±0.07^c^ | 11.60±0.49^b^ | 27.66±1.09^a^ | 24.85±2.10^a^ | 13.72±0.98^b^ | 12.05±0.29^b^ |
| 14 | ethyl tetradecanoate | 47.32±1.23^c^ | 42.39±0.78^d^ | 55.10±4.08^b^ | 77.88±0.18^a^ | 38.95±1.31^e^ | 41.66±0.18^d^ |
| 15 | ethyl pentadecanoate | 7.06±0.35^b^ | 5.89±0.28^c^ | 7.00±0.46^b^ | 9.14±0.28^a^ | 4.27±0.16^d^ | 3.38±0.53^e^ |
| 16 | ethyl hexadecanoate | 87.05±3.96^bc^ | 91.24±3.13^b^ | 131.37±15.62^a^ | 142.11±13.35^a^ | 86.34±1.78^c^ | 93.39±0.23^b^ |
| 17 | ethyl 9-hexadecenoate | 5.99±0.11^b^ | 5.61±0.05^c^ | 5.81±0.21^bc^ | 6.62±0.11^a^ | 3.57±0.03^e^ | 4.24±0.11^d^ |
| 18 | ethyl 15-methylpentadecanoate | 0.58±0.07^a^ | 0.43±0.04^bc^ | 0.55±0.00^a^ | 0.39±0.10^c^ | 0.46±0.04^b^ | 0.26±0.09^d^ |
| 19 | ethyl oleate | 16.12±1.15^a^ | 13.73±1.16^b^ | 14.72±0.54^ab^ | 15.62±2.24^ab^ | 11.02±0.12^c^ | 7.91±1.63^d^ |
| 20 | ethyl linoleate | 21.56±1.10^a^ | 19.68±2.06^b^ | 21.23±1.12^a^ | 10.71±2.50^cd^ | 11.19±0.05^c^ | 8.05±1.84^d^ |
| 21 | ethyl linolenate | 0.61±0.05^a^ | 0.66±0.15^a^ | 0.63±0.04^a^ | 0.23±0.06^b^ | 0.18±0.02^bc^ | 0.13±0.02^c^ |
| 22 | propyl hexanoate | 2.93±0.09^b^ | 3.94±0.04^a^ | 0.45±0.05^d^ | 2.67±0.17^b^ | 1.08±0.06^c^ | 1.24±0.25^c^ |
| 23 | butyl hexanoate | 17.84±0.83^b^ | 30.66±2.63^a^ | 4.96±0.29^d^ | 29.51±0.44^a^ | 13.45±1.49^c^ | 15.75±3.31^bc^ |
| 24 | isopentyl hexanoate | 18.57±0.32^c^ | 20.57±1.29^b^ | 19.70±1.68^bc^ | 26.57±2.28^a^ | 13.76±1.72^d^ | 13.08±2.89^d^ |
| 25 | pentyl hexanoate | 12.93±0.30^d^ | 28.24±1.31^a^ | 4.92±0.28^f^ | 26.60±1.45^b^ | 9.32±0.04^e^ | 16.31±3.66^c^ |
| 26 | isopentyl methoxyacetate | 2.16±0.41^b^ | 2.09±0.33^b^ | 10.34±1.35^a^ | 2.33±0.00^b^ | 2.21±0.12^b^ | 1.40±0.19^c^ |
| 27 | hexyl hexanoate | 78.11±1.79^d^ | 112.01±1.70^a^ | 62.97±3.68^e^ | 97.24±3.17^c^ | 94.82±1.75^c^ | 107.58±4.58^b^ |
| 28 | isopentyl octanoate | 2.44±0.06^b^ | 1.54±0.02^c^ | 2.42±0.25^b^ | 2.85±0.01^a^ | 1.56±0.24^c^ | 1.37±0.34^c^ |
| 29 | furfuryl hexanoate | 1.71±0.11^e^ | 4.54±0.12^a^ | 3.45±0.54^c^ | 4.47±0.16^a^ | 2.20±0.17^d^ | 4.13±0.06^b^ |
| 30 | phenylethyl butyrate | 2.29±0.11^c^ | 4.27±0.10^a^ | 3.83±0.13^b^ | 4.61±0.14^a^ | 2.30±0.10^c^ | 2.53±0.32^c^ |
| **Acids (6)** | | | | | | | |
| 31 | butanoic acid | 2.49±0.45^b^ | 2.47±0.35^b^ | 7.64±0.06^a^ | 2.12±0.01^bc^ | 1.09±0.07^d^ | 1.96±0.01^c^ |
| 32 | 3-methylbutanoic acid | 1.19±0.26^c^ | 2.65±0.58^a^ | 2.79±0.01^a^ | 0.61±0.00^d^ | 0.34±0.08^e^ | 1.74±0.07^b^ |
| 33 | pentanoic acid | 5.22±0.25^b^ | 7.74±1.12^a^ | 6.03±0.09^a^ | 6.50±0.29^a^ | 4.00±0.41^c^ | 3.29±0.27^d^ |
| 34 | hexanoic acid | 62.67±1.27^b^ | 60.35±2.83^b^ | 58.00±4.33^b^ | 73.02±2.68^a^ | 36.77±1.53^d^ | 47.48±1.23^c^ |
| 35 | heptanoic acid | 3.15±0.33^c^ | 8.23±0.13^a^ | 5.16±0.34^b^ | 4.45±0.87^b^ | 2.51±0.11^d^ | 8.57±2.53^a^ |
| 36 | octanoic acid | 6.09±0.56^e^ | 9.58±0.09^d^ | 16.09±1.76^a^ | 14.62±2.26^b^ | 11.39±0.58^c^ | 15.68±2.18^a^ |
| **Alcohols (4)** | | | | | | | |
| 37 | 1-butanol | 9.07±0.21^a^ | 4.38±0.29^c^ | 3.33±0.17^d^ | 3.23±0.17^d^ | 5.45±0.16^b^ | 2.61±0.09^e^ |
| 38 | 3-methyl-1-butanol | 10.40±2.32^a^ | 5.24±0.12^c^ | 9.94±0.50^a^ | 9.84±0.50^a^ | 7.57±0.05^b^ | 2.39±0.20^d^ |
| 39 | 1-hexanol | 21.25±0.74^d^ | 39.44±0.21^a^ | 32.52±1.38^b^ | 32.27±1.28^b^ | 24.53±0.83^c^ | 23.68±0.01^c^ |
| 40 | benzeneethanol | 0.92±0.03^b^ | 0.86±0.04^b^ | 3.67±0.45^a^ | 3.71±0.45^a^ | 0.63±0.00^c^ | 0.22±0.01^d^ |
| **Ketone (4)** | | | | | | | |
| 41 | 2-nonen-4-one | 1.16±0.05^e^ | 1.98±0.05^d^ | 3.71±0.05^b^ | 4.68±0.26^a^ | 2.70±0.24^c^ | 3.46±0.28^b^ |
| 42 | 2-undecanone | 1.72±0.04^c^ | 1.35±0.02^d^ | 1.96±0.26^b^ | 2.88±0.05^a^ | 1.41±0.17^d^ | 1.91±0.07^b^ |
| 43 | 2-pentadecanone | 1.20±0.01^c^ | 1.36±0.04^b^ | 2.02±0.01^a^ | 2.40±0.21^a^ | 0.68±0.15^d^ | 0.94±0.30^d^ |
| 44 | 6,10,14-2-pentadecanone | 1.14±0.02^c^ | 1.33±0.04^b^ | 1.07±0.06^c^ | 1.70±0.11^a^ | 0.73±0.19^d^ | 0.67±0.02^d^ |
| **Aromatics (3)** | | | | | | | |
| 45 | 4-methylphenol | 0.55±0.04^d^ | 2.03±0.18^a^ | 1.68±0.14^b^ | 1.70±0.10^b^ | 0.99±0.14^c^ | 1.54±0.40^bc^ |
| 46 | 2,4-di-ter-butyl-phenol | 7.91±0.04^b^ | 7.55±0.32^b^ | 12.59±0.70^a^ | 2.99±0.11^c^ | 2.36±0.03^d^ | 2.25±0.07^d^ |
| 47 | (2,2-diethoxyethyl)-benzene | 2.29±0.04^d^ | 2.49±0.30^d^ | 25.34±2.50^a^ | 3.16±0.21^c^ | 4.39±0.42^b^ | 2.36±0.29^d^ |
| **Aldehydes (2)** | | | | | | | |
| 48 | furfural | 4.07±0.04^e^ | 4.77±0.29^d^ | 14.62±0.75^b^ | 17.07±0.55^a^ | 8.66±0.22^c^ | 2.43±0.16^f^ |
| 49 | benzaldehyde | 2.55±0.09^d^ | 3.88±0.15^c^ | 7.88±0.49^a^ | 5.06±0.63^b^ | 4.39±0.22^bc^ | 4.34±0.15^bc^ |

Different letters obtained by ANOVA indicate significant differences at *p* < 0.05 (n = 3).
